# Determinants of functional cure in interferon-treated chronic hepatitis B: a retrospective cohort analysis of HBsAg dynamics and clinical predictors

**DOI:** 10.3389/fcimb.2025.1615327

**Published:** 2025-06-20

**Authors:** Daqiong Zhou, Jianru Jia, Feng Zhao, Jiangyu Liu, Zichen Zhang, Zhenhuan Cao

**Affiliations:** ^1^ Beijing Youan Hospital, Capital Medical University, Beijing, China; ^2^ Liver Disease Center of Baoding People's Hospital, Hebei, China; ^3^ Laboratory for Clinical Medicine, Capital Medical University, Beijing, China

**Keywords:** CHB, chronic HBV infection, PEG-IFN, pegylated interferon, FC, functional cure, HBV - hepatitis B virus, HBsAg clearance

## Abstract

**Background and Objectives:**

Functional cure of chronic hepatitis B (CHB) is the optimal goal of antiviral therapy. This study evaluates the functional cure rates in a large cohort treated with interferon (IFNα) and explores baseline predictors to optimize personalized treatment strategies.

**Methods:**

This retrospective study included CHB patients treated with IFN monotherapy or IFN combined with nucleos(t)ide analogs at Beijing You’an Hospital from 2008 to 2023. Patients were categorized into functional cure (FC) and non-functional cure (NFC) groups based on treatment outcomes.

**Results:**

Among 5288 patients, 887 (16.8%) achieved functional cure. FC patients had significantly lower baseline HBsAg, HBV DNA, and HBeAg positivity compared to NFC patients. Kaplan-Meier analysis revealed cumulative functional cure rates of 10.6%, 25.7%, and 39.9% at 48, 96, and 144 weeks, respectively. Multivariate logistic regression analysis identified lower baseline HBsAg levels, female, HBeAg negativity, lower γ-GT levels, and higher FT3 and FT4 levels as factors associated with higher functional cure rates. Patients with HBsAg <100 IU/ml had a cure rate of 58.8% within 96 weeks, compared to 11.1% for those with >1500 IU/ml.

**Conclusion:**

Early HBsAg decline is critical for functional cure. Female patients, HBeAg-negative status, and baseline HBsAg <100 IU/mL predict higher success rates.

## 1 Introduction

According to the latest estimates from the World Health Organization, the global seroprevalence of hepatitis B surface antigen (HBsAg) in 2019 was 4.1%, with approximately 316 million people living with chronic hepatitis B (CHB) (Global, regional, and national burden of hepatitis B, 1990-2019: a systematic analysis for the Global Burden of Disease Study 2019, 2022). HBV infection is a major contributor to the development of liver cirrhosis and hepatocellular carcinoma (HCC). Additionally, acute exacerbations, viral reactivation, extrahepatic complications, and psychosocial impacts further exacerbate the societal burden of HBV infection (Hsu et al., 2023). Early diagnosis, appropriate antiviral therapy, and proactive monitoring of clinical complications are critical to alleviating the disease burden in individuals with HBV infection. Currently, the optimal therapeutic goal for CHB is achieving functional cure, but this target remains challenging to achieve on a broad scale (Ghany et al., 2023).

Interferon (IFN) has been used to treat CHB for nearly 50 years and has demonstrated higher rates of HBeAg and HBsAg seroclearance compared to nucleos(t)ide analogs (NAs) (Wong et al., 2022). In recent years, various combination strategies involving NAs and IFNα have been evaluated to improve HBsAg seroclearance rates. A meta-analysis of 60 studies revealed that combination therapy with NAs and IFNα significantly enhances HBsAg seroclearance rates compared to NA monotherapy (Liu et al., 2020). Recent studies further indicate that IFNα, as an add-on therapy, can improve HBsAg clearance rates in HBeAg-negative CHB patients who have been treated with NAs for more than one year (Wu et al., 2020; Farag et al., 2024).

Numerous studies have highlighted the importance of baseline biomarkers, including HBsAg levels, HBV DNA levels, HBeAg status, anti-HBc, and liver function, in predicting patient responses to antiviral therapy (Chan et al., 2018; Wang et al., 2022; Wang et al., 2024; Zhang et al., 2024). Generally, patients with undetectable HBV DNA, HBeAg negativity, and lower baseline HBsAg levels are considered ideal candidates for achieving clinical cure with IFNα therapy. However, in real-world clinical practice, interferon is used not only for these favorable patient populations but also for CHB patients with varying baseline characteristics. The differences in HBsAg seroclearance rates and the dynamics of HBsAg levels during IFNα treatment among favorable and non-favorable patients remain poorly understood. The primary reasons include the relatively limited use of interferon compared to nucleos(t)ide analogs due to its significant adverse effects and the inherent challenges of achieving functional cure for hepatitis B. Consequently, previous studies on functional cure have been limited, with few large-scale investigations addressing this critical outcome.

This study systematically analyzed data from a large cohort of CHB patients treated with interferon to evaluate the relationships between various baseline characteristics and functional cure. Additionally, generalized estimating equations were applied to assess the overall trend of HBsAg level changes. These findings aim to provide more precise individualized assessment and treatment guidance for CHB patients.

## 2 Patients and methods

### 2.1 Study population

**Figure 1:**
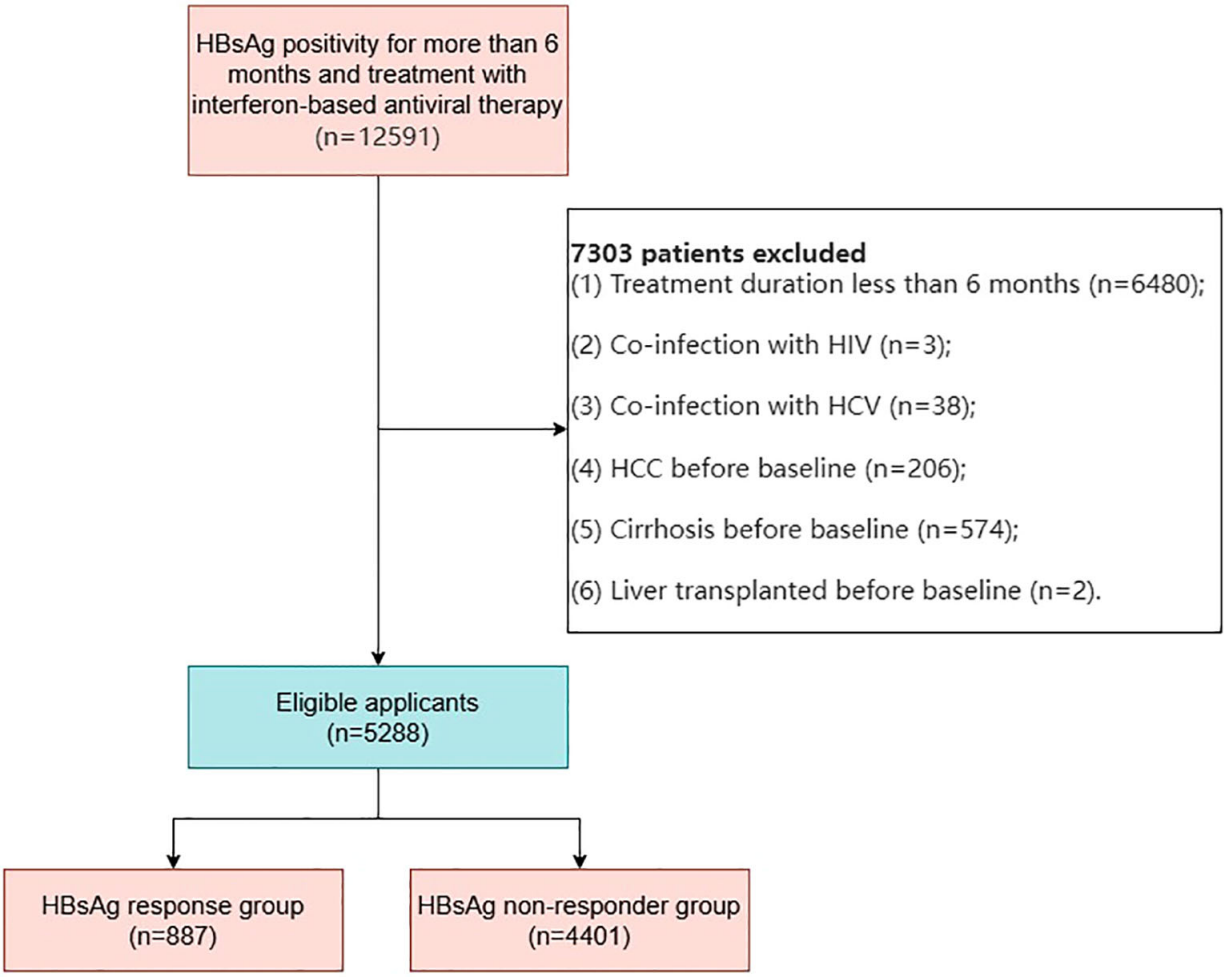
Flowchart of patient selection for chronic hepatitis B patients receiving interferon therapy.

We collected data from CHB patients who visited Beijing You’an Hospital between February 2008 and January 2023. All patients had persistent HBsAg positivity for more than six months and were treated with standard interferon or pegylated interferon, either alone or in combination with one or more nucleos(t)ide analogs, including lamivudine, adefovir dipivoxil, telbivudine, tenofovir disoproxil fumarate, tenofovir alafenamide, or amitibivir (**Figure 1**).

The study protocol was approved by the Clinical Research Ethics Committee of Beijing You’an Hospital, Capital Medical University.

### 2.2 Data collection

Data were retrieved in April 2023 from the outpatient system of Beijing You’an Hospital. The baseline date was defined as the first recorded date of IFN treatment initiation. Demographic data, including birth date and gender, were collected along with serial measurements of liver and renal biochemistry, hematology, and HBV virological markers (HBV DNA, HBsAg, HBsAb, HBeAg, and HBeAb) during IFN treatment and follow-up. Imaging results, including ultrasound, CT, MRI, and endoscopic findings, were also collected.

The lower limit of quantification for HBsAg was 0.05 IU/mL. Anti-HBs positivity was defined as >10 IU/mL, HBeAg negativity as <1 COI, and Anti-HBe negativity as >1 COI. HBV DNA was measured using high-sensitivity assays with a detection limit of 20 IU/mL.

### 2.3 Endpoints and definitions

Functional cure was defined as HBsAg negativity in the serum confirmed by at least two consecutive tests with a minimum interval of 12 weeks, with or without the presence of anti-HBs, accompanied by HBeAg negativity and HBV DNA levels below the detection limit during the post-treatment follow-up period. The follow-up endpoint for patients achieving HBsAg seroclearance was the first recorded date of seroclearance, while for patients who did not achieve HBsAg seroclearance, the endpoint was the date of the last follow-up.

### 2.4 Statistical analysis

Continuous variables were expressed as medians with interquartile ranges (IQR) and compared between groups using the Mann-Whitney U test. Categorical variables were expressed as percentages and analyzed using the chi-square test or Fisher’s exact test for group differences.

The trends in HBsAg levels were analyzed using a linear mixed-effects model, incorporating random effects for patients to account for inter-individual variability. Fixed effects included time (in months), group classification (functional cure group *vs*. non-cure group; HBsAg <100 *vs*. 100–1500 *vs*. >1500 IU/mL; HBeAg-positive *vs*. HBeAg-negative; male *vs*. female), and the interaction between time and group classification. Marginal effects of HBsAg level changes over time across groups were estimated using generalized estimating equations (GEE). A Loess local regression model was applied to assess the temporal trends of HBsAg levels for each group, and differences between groups were visualized graphically. The cumulative incidence of HBsAg seroclearance was estimated using the Kaplan-Meier method, and subgroup differences were compared using the log-rank test.

Multiple imputation (MI) was employed to handle missing data across all variables. Multiple imputation is a robust statistical method that fills in missing values by using the relationships between observed data points. This approach creates several (typically 5–20) imputed datasets, which are then analyzed separately, and the results are combined to provide estimates that account for the uncertainty due to missing values. This method helps reduce potential bias that may arise from simply excluding missing data and improves the overall robustness of our model by utilizing all available information. **Supplementary Table 1** summarizes the missingness for each variable. Both univariate and multivariate logistic regression analyses were performed on the imputed dataset to evaluate the associations between baseline demographic characteristics, hematological markers, HBV virological markers, and functional cure.

Data analysis and visualization were conducted using R software (version 4.2.0). All statistical tests were two-sided, with a significance threshold of P<0.05.

## 3 Results

### 3.1 Baseline clinical characteristics

In this study, 12,591 CHB patients with persistent HBsAg positivity for more than six months who received interferon-based antiviral therapy, with or without nucleos(t)ide analogs, were initially identified. After rigorous screening, 5,288 patients met the inclusion criteria and were included in the study. These patients were categorized into two groups (**Figure 1**): Functional Cure (FC) Group (n=887): Patients who achieved functional cure during follow-up; Non-Functional Cure (NFC) Group (n=4,401): Patients who did not achieve functional cure during follow-up.

The baseline clinical characteristics of patients in the FC and NFC groups showed significant differences across several parameters (**Table 1**). The proportion of males was higher in the NFC group compared to the FC group (66.1% *vs*. 62.2%, P=0.015). Baseline levels of HBsAg (IQR: 2.27 *vs*. 3.38 log10 IU/mL, P<0.001), HBV DNA (IQR: 0 *vs*. 2.75 log10 IU/mL, P<0.001), and HBeAg positivity (38% *vs*. 63.5%, P<0.001) were significantly lower in the FC group compared to the NFC group.

**Table 1:** Baseline clinical characteristics of functional cure (FC) and non-cure (NFC) patients.

Characteristics	Total (n=5288)	FC (n=887)	NFC (n=4401)	P-value
Sex (Male)	65.5%	62.2%	66.1%	0.015
Age(years)	31(26,39)	32(26,39)	31(26,38)	0.494
IFN+NAs (%)	81.1%	79.5%	81.4%	0.104
HBsAg (log_10_IU/mL)	3.26(2.53,3.93)	2.27(1.37,3.05)	3.38(2.80,4.00)	<0.001
HBV DNA (log_10_IU/mL)	2.48(0,5.53)	0(0,3.45)	2.75(1.37,5.98)	<0.001
HBeAg positivity rate (%)	59.2%	38.0%	63.5%	<0.001
Hepatic steatosis (%)	50.7%	52.4%	50.3%	0.135
ALT (U/L)	44.1(24.0,93.2)	36.0(20.3,69.1)	46.4(25.0,98.1)	<0.001
AST (U/L)	34.8(24.2,59.3)	29.0(22.1,47.7)	36.1(24.9,62.0)	<0.001
γ-GT (U/L)	25.0(16.0,43.5)	21.4(14.5,33.9)	26.0(16.5,45.7)	<0.001
FT3 (pmol/L)	4.7(4.1,5.1)	4.7(4.2,5.2)	4.7(4.1,5.1)	0.027
FT4 (pmol/L)	13.6(12.2,15.2)	13.7(12.3,15.4)	13.6(12.1,15.2)	0.035
TSH (mIU/L)	1.6(1.1,2.3)	1.6(1.1,2.3)	1.6(1.1,2.3)	0.694
WBC (*10^9/L)	5.1(4.0,6.3)	5.1(3.9,6.2)	5.1(4.1,6.3)	0.150
ANC (*10^9/L)	2.7(2.0,3.7)	2.7(2.0,3.7)	2.7(2.0,3.6)	0.815
Plt (*10^9/L)	186(144,226)	187(141,224)	186(145,226)	0.723

ALT, alanine aminotransferase; AST, aspartate aminotransferase; γ-GT, γ-glutamyl transpeptidase; FT3, free triiodothyronine; FT4, free thyroxine; TSH, thyroid-stimulating hormone; WBC, white blood cell; ANC, absolute neutrophil count; Plt, platelet.

Additionally, although the median values of thyroid function markers were within the normal range in both groups, the FC group exhibited slightly higher levels of FT3 and FT4 compared to the NFC group (FT3: P=0.027, FT4: P=0.035).

### 3.2 HBsAg serum kinetics and functional cure rates

This study collected longitudinal HBsAg data from 5,288 chronic hepatitis B (CHB) patients treated with interferon, resulting in a total of 54,582 HBsAg data points. The serum kinetics of HBsAg levels over time and the cumulative functional cure rates were analyzed.

**Figure 2:**
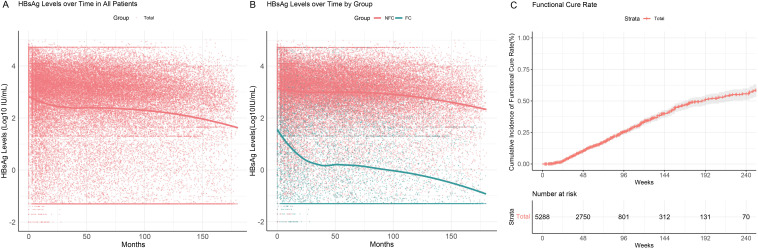
HBsAg serum kinetics and cumulative functional cure rates in chronic hepatitis B patients. **(A)** HBsAg serum kinetics in the overall patient population; **(B)** HBsAg serum kinetics in the FC and NFC groups; **(C)** Cumulative functional cure rates.

During follow-up, a notable decline in HBsAg levels was observed within the first two years (**Figure 2A**), particularly in the FC group (green), where HBsAg levels demonstrated a rapid decrease within the initial 25 months of treatment. In contrast, HBsAg levels in the NFC group (red) showed no significant changes, even with prolonged follow-up (**Figure 2B**). These findings suggest that HBsAg seroclearance is closely associated with early treatment response, indicating a higher likelihood of cure or better treatment outcomes for responsive patients. Conversely, patients in the NFC group may require further evaluation and potential treatment adjustments.

All 5,288 patients included in the study were free of liver cirrhosis and hepatocellular carcinoma at baseline. The median duration of interferon therapy was 48 weeks (IQR: 32–76 weeks), with a median follow-up duration of 3.4 years (IQR: 1.3–8.0 years). A total of 887 patients (887/5288, 16.8%) achieved functional cure. The cumulative functional cure rates were 10.6% (95% CI: 9.6–11.5%) at 48 weeks, 25.7% (95% CI: 23.8–27.7%) at 96 weeks, and 39.9% (95% CI: 37.0–42.9%) at 144 weeks (**Figure 2C**).

### 3.3 Factors associated with functional cure

Univariate logistic regression analysis identified gender, baseline HBsAg levels, HBV DNA levels, HBeAg positivity, ALT, AST, γ-GT, FT3, and FT4 as significant factors influencing functional cure (**Table 2**). Multivariate analysis, which adjusted for these variables, revealed the following statistically significant associations: Male gender (OR: 0.754, 95% CI: 0.634–0.897, P=0.001), Baseline HBsAg level (OR: 0.480, 95% CI: 0.445–0.517, P<0.001), HBeAg status (OR: 0.825, 95% CI: 0.688–0.988, P=0.037), γ-GT (OR: 0.997, 95% CI: 0.994–1.000, P=0.022), FT3 (OR: 1.134, 95% CI: 1.010–1.273, P=0.033), FT4 (OR: 1.028, 95% CI: 1.003–1.055, P=0.030). These findings suggest that female gender, lower baseline levels of HBsAg and GGT, HBeAg negativity, and higher thyroid function markers (FT3 and FT4) are associated with achieving functional cure.

**Table 2:** Factors associated with functional cure.

Characteristics	Univariate analysis		Multivariable analysis	
	OR (95%CI)	P value	OR (95%CI)	P value
Sex (Male)	0.844(0.727-0.980)	0.026	0.754(0.634-0.897)	0.001
Age(years)	1.002(0.995-1.010)	0.569		
IFN+NAs	0.887(0.741-1.062)	0.202		
HBsAg (log_10_IU/mL)	0.475(0.446-0.506)	<0.001	0.480(0.445-0.517)	<0.001
HBV DNA (log_10_IU/mL)	0.873(0.849-0.897)	<0.001	1.012(0.979-1.047)	0.478
HBeAg (+)	0.353(0.304-0.409)	<0.001	0.825(0.688-0.988)	0.037
Hepatic steatosis	1.087(0.941-1.256)	0.255		
ALT (U/L)	0.998(0.998-0.999)	<0.001	1.001(1.000-1.003)	0.108
AST (U/L)	0.997(0.995-0.998)	<0.001	0.998(0.995-1.000)	0.099
γ-GT (U/L)	0.995(0.993-0.997)	<0.001	0.997(0.994-1.000)	0.022
FT3 (pmol/L)	1.115(1.037-1.198)	0.003	1.134(1.010-1.273)	0.033
FT4 (pmol/L)	1.032(1.016-1.049)	<0.001	1.028(1.003-1.055)	0.030
TSH (mIU/L)	0.994(0.977-1.012)	0.518		
WBC (*10^9/L)	0.979(0.939-1.021)	0.330		
ANC (*10^9/L)	1.020(0.970-1.072)	0.450		
Plt (*10^9/L)	1.000(0.999-1.001)	0.812		

ALT, alanine aminotransferase; AST, aspartate aminotransferase; γ-GT, γ-glutamyl transpeptidase; FT3, free triiodothyronine; FT4, free thyroxine; TSH, thyroid-stimulating hormone; WBC, white blood cell; ANC, absolute neutrophil count; Plt, platelet.

### 3.4 Functional cure rates across different baseline HBsAg levels

Based on the results of multivariate logistic regression analysis, we performed stratified analyses to compare HBsAg serum kinetics and cumulative functional cure rates in CHB patients with different baseline HBsAg levels (<100 IU/mL, 100–1500 IU/mL, >1500 IU/mL) during interferon treatment and follow-up.

**Figure 3:**
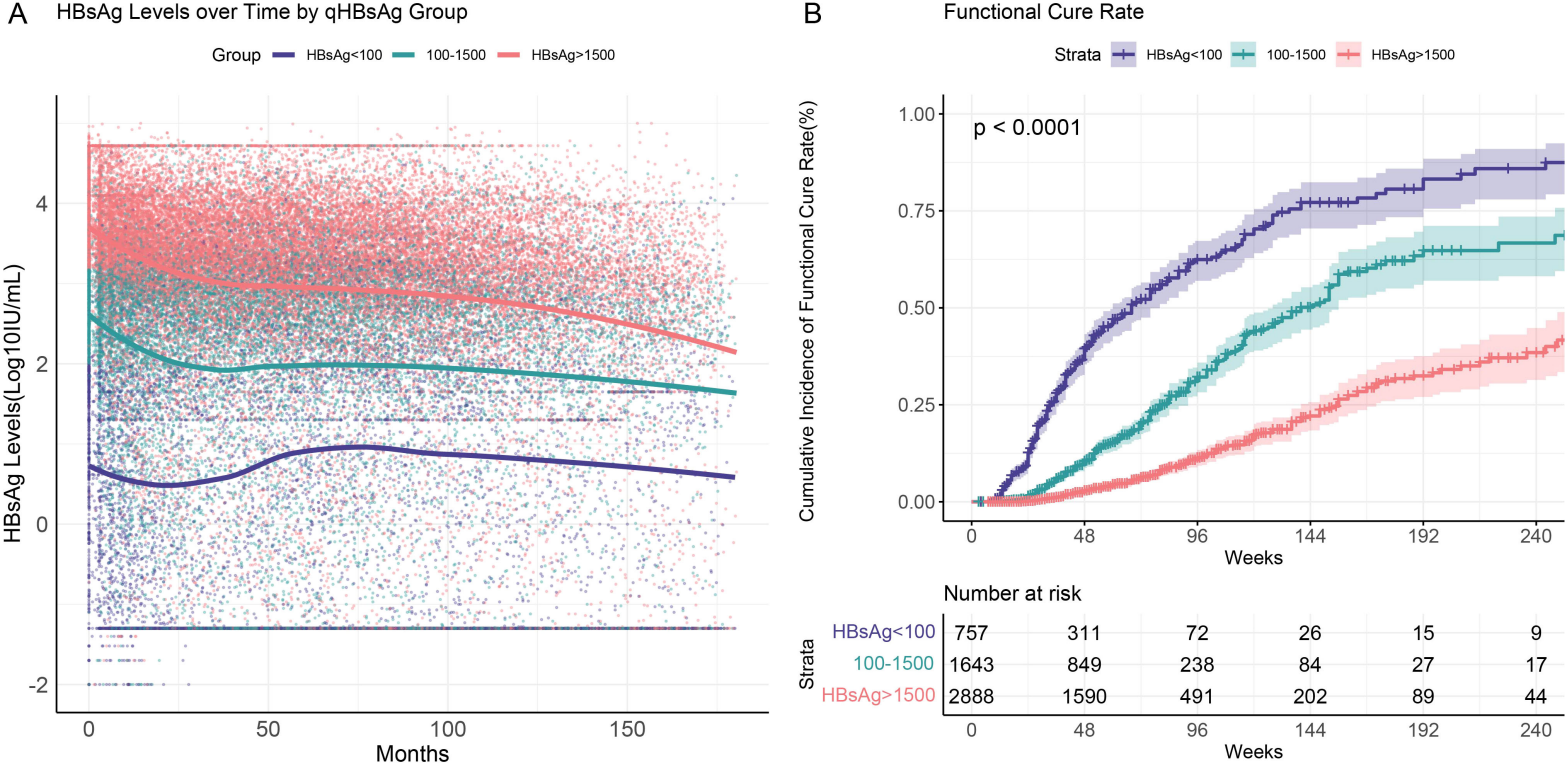
HBsAg serum kinetics and cumulative functional cure rates in chronic hepatitis B patients with different baseline HBsAg levels (<100 IU/mL, 100–1500 IU/mL, >1500 IU/mL). **(A)** HBsAg serum kinetics; **(B)** Cumulative functional cure rates.

For all patients, HBsAg levels showed a rapid decline within the first 25 months, coinciding with the active interferon treatment phase, after which the rate of decline plateaued (**Figure 3A**). This trend was particularly evident in patients with higher baseline HBsAg levels (100–1500 IU/mL and >1500 IU/mL). Notably, CHB patients with low baseline HBsAg levels (<100 IU/mL) who failed to achieve functional cure within the first 25 months after initiating interferon therapy experienced a rebound in HBsAg levels to baseline or even higher levels upon cessation of therapy. In 757 patients with chronic hepatitis B and baseline HBsAg levels below 100 IU/mL, a total of 425 (56%) patients had one or more HBsAg quantification measurements above baseline levels during follow-up. Among the patients who experienced a rebound, 227 (53.4%) achieved the criteria for functional cure during the follow-up period. Within 96 weeks of interferon treatment, patients with baseline HBsAg levels <100 IU/mL achieved a cumulative functional cure rate of 58.8% (95% CI: 53.9%–63.8%), compared to 34.0% (95% CI: 30.2%–38.1%) for those with levels between 100 and 1500 IU/mL, and only 11.1% (95% CI: 9.3%–13.3%) for those with levels >1500 IU/mL. Differences in functional cure rates among the groups were statistically significant (P<0.001) based on the Log-rank test (**Figure 3B**).

Therefore, for patients with low baseline HBsAg levels, although early treatment shows some efficacy, premature discontinuation or failure to maintain appropriate therapy may lead to a decline in immune response, resulting in viral rebound. In these patients, continued monitoring during the later stages of treatment and extended treatment periods may lead to better outcomes. Thus, for patients who are unable to maintain low HBsAg levels without meeting the criteria for functional cure, continued follow-up is necessary, with treatment duration extended based on their response.

### 3.5 Functional cure rates across different HBeAg statuses

**Figure 4:**
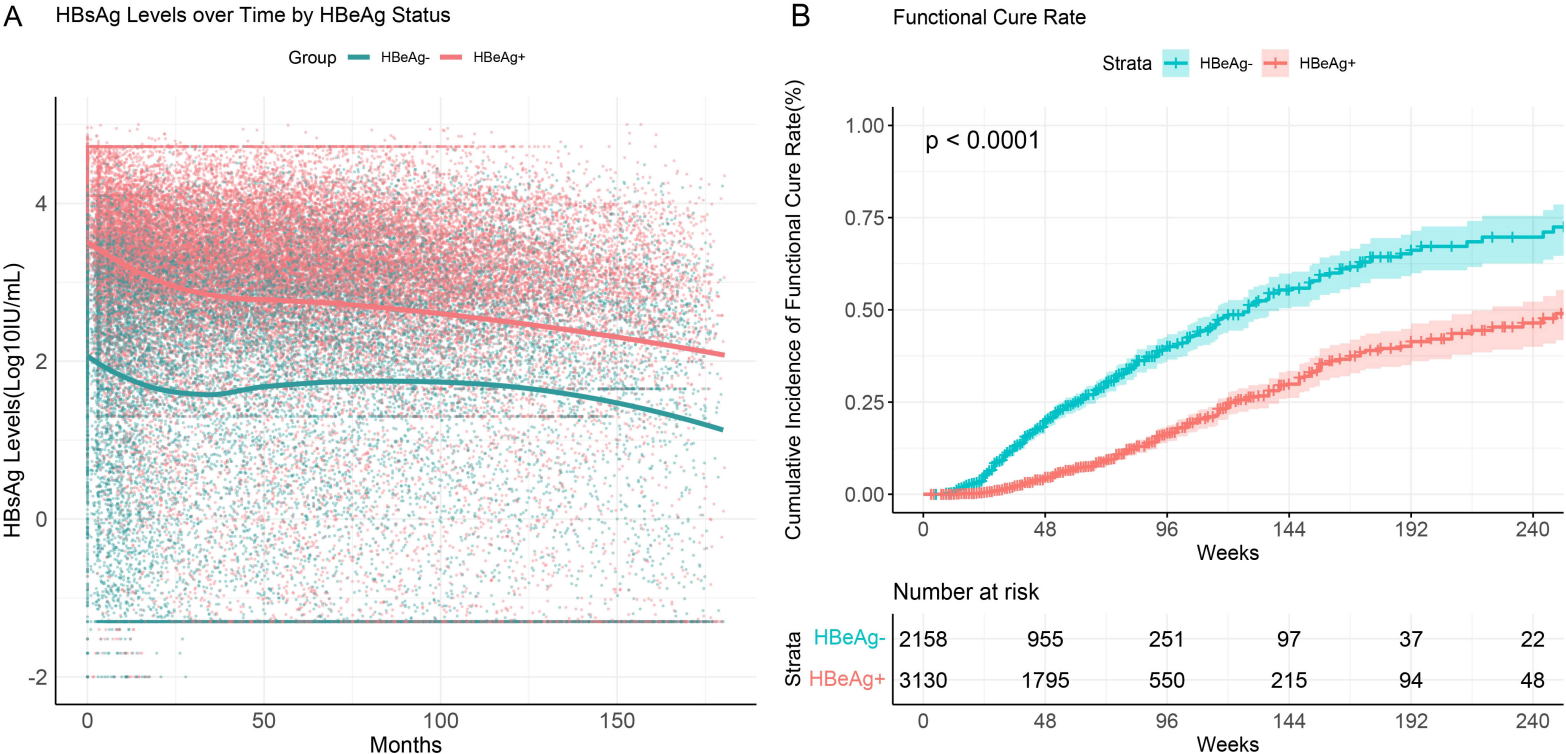
HBsAg serum kinetics and cumulative functional cure rates in chronic hepatitis B patients with different HBeAg statuses. **(A)** HBsAg serum kinetics; **(B)** Cumulative functional cure rates.

We performed subgroup analyses based on baseline HBeAg status. The results showed that throughout the follow-up period, HBeAg-positive patients consistently exhibited higher HBsAg levels compared to HBeAg-negative patients (**Figure 4A**). HBeAg-positive patients demonstrated a significant decline in HBsAg levels within the first 25 months of initiating interferon therapy, whereas HBeAg-negative patients exhibited a less pronounced decline. During the post-treatment follow-up phase, the decline in HBsAg levels slowed and eventually stabilized.

The functional cure rate was significantly higher in HBeAg-negative patients compared to HBeAg-positive patients. Among CHB patients who were HBeAg-negative at baseline, the cumulative functional cure rate within 96 weeks was 40.1% (95% CI: 36.8%–43.6%), whereas for HBeAg-positive patients, it was only 16.6% (95% CI: 14.5%–18.9%), with the difference being statistically significant (P<0.001) (**Figure 4B**).

These findings suggest that HBeAg status plays a critical role in determining HBsAg dynamics and functional cure rates in CHB patients. HBeAg-negative patients with lower baseline HBsAg levels demonstrated a more pronounced response to interferon therapy, as reflected by higher functional cure rates.

### 3.6 Functional cure rates across different sexes

**Figure 5:**
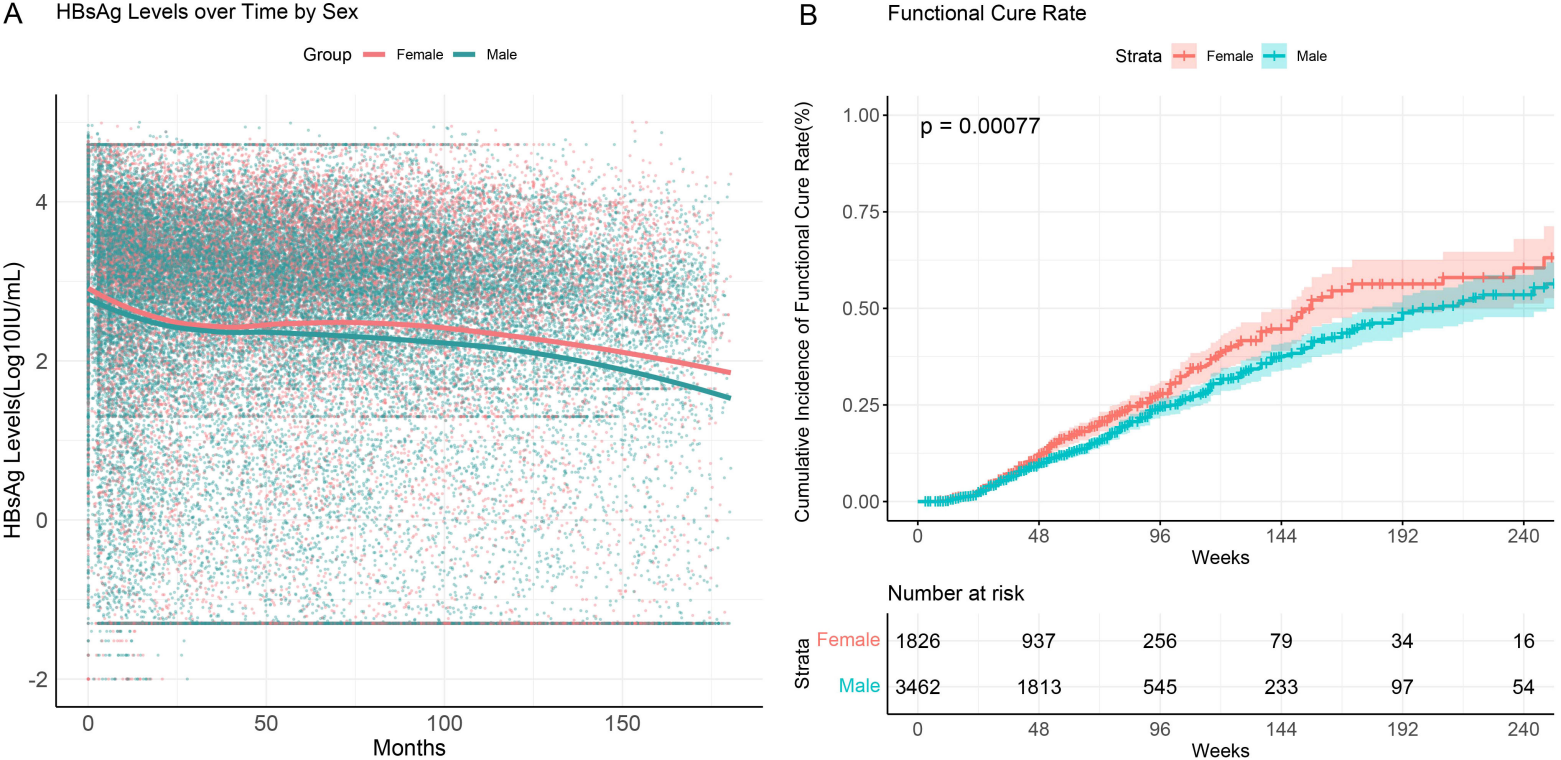
HBsAg serum kinetics and cumulative functional cure rates in chronic hepatitis B patients across different sexes. **(A)** HBsAg serum kinetics; **(B)** Cumulative functional cure rates.

Multivariate logistic regression analysis indicated a correlation between sex and functional cure. To further investigate this, we analyzed differences in HBsAg serum kinetics and cumulative functional cure rates between male and female patients (**Figure 5**). The results showed that while the overall trends in HBsAg level changes during follow-up were similar between sex, female patients exhibited significantly higher cumulative functional cure rates compared to male patients. At 96 weeks of interferon treatment, the cumulative functional cure rate was 28.0% (95% CI: 24.8%–31.5%) for females and 24.5% (95% CI: 22.2%–26.9%) for males, with the difference being statistically significant (P=0.00077).

These findings suggest a significant gender-based difference in functional cure among CHB patients. Female patients demonstrated higher functional cure rates during both the early phase of treatment and long-term follow-up, indicating that females may have greater sensitivity to treatment or a stronger immune response to therapy.

## 4 Discussion

This study retrospectively analyzed functional cure outcomes in 5,288 chronic hepatitis B (CHB) patients receiving interferon therapy, highlighting the impact of various baseline characteristics and biochemical markers on treatment efficacy. To the best of our knowledge, this is the largest real-world study to date focusing on CHB patients treated with interferon, particularly with functional cure as the therapeutic goal. The findings of this study provide valuable insights and serve as an important reference for individualized treatment strategies in CHB patients.

First, the critical role of baseline clinical characteristics in achieving functional cure deserves attention. Baseline HBsAg levels are instrumental in distinguishing immune tolerance from immune clearance in HBeAg-positive patients, predicting inactive disease and spontaneous HBsAg seroclearance in HBeAg-negative patients, and are essential for individualizing pegylated interferon (Peg-IFN) therapy (Cornberg et al., 2017). In this study, patients in the FC group had significantly lower baseline HBsAg and HBV DNA levels compared to the NFC group, along with a lower HBeAg positivity rate, consistent with findings from previous studies (Chuaypen et al., 2018; Mimura et al., 2021; Chu et al., 2022). CHB patients with lower baseline HBsAg levels and HBeAg negativity demonstrated higher functional cure rates following interferon therapy, suggesting that baseline HBsAg levels and HBeAg status are important predictive factors. These results highlight the importance of risk stratification prior to treatment, as patients with lower baseline levels may derive greater benefit from interferon-based therapy.

Second, the analysis of HBsAg trends over time revealed that patients in the FC group experienced a significant decline in HBsAg levels during the early phase of treatment, whereas the NFC group showed relatively minor changes. This underscores the critical importance of early treatment response in determining long-term outcomes. Previous prospective research by our team demonstrated that quantitative HBsAg levels and changes during the early phase of treatment (Weeks 12 and 24) are strong predictors of HBsAg seroclearance (Cao et al., 2017). Similarly, a study by Filip De Ridder et al. reported that the mean decline in HBsAg levels from baseline at Week 24 serves as an early marker for HBsAg clearance during Peg-IFN therapy (De Ridder et al., 2021). Even among untreated patients, a rapid decline in HBsAg levels has been associated with higher rates of spontaneous HBsAg seroclearance (Chen et al., 2012; Lin et al., 2023). These findings suggest that early changes in HBsAg levels provide critical insights into subsequent treatment responses. This highlights the clinical utility of early HBsAg monitoring as an effective biomarker for predicting functional cure. For patients demonstrating suboptimal treatment responses, trends in HBsAg levels during the early phase can guide treatment modifications to improve success rates. An interesting phenomenon observed in this study is that while all patients exhibited a rapid decline in HBsAg levels during interferon therapy, distinct post-treatment dynamics were noted. Patients with higher baseline HBsAg levels (100–1500 IU/mL and >1500 IU/mL) continued to show a steady decline in HBsAg levels after stopping interferon therapy. In contrast, patients with lower baseline HBsAg levels (<100 IU/mL) who failed to achieve functional cure experienced a rebound in HBsAg levels to baseline or even higher levels after treatment cessation. Therefore, the results of this study suggest that for patients who fail to achieve functional cure under standard IFN therapy, particularly those with low baseline HBsAg levels who are unable to maintain a sustained clearance state, it becomes crucial to adjust the treatment strategy. While IFN therapy is widely used in hepatitis B, its efficacy varies across different patient populations. With the continuous development of next-generation antiviral drugs, such as PD-1 receptor antagonists and small molecules, these treatments may become part of an optimized therapeutic regimen. For patients with persistent or rebounding HBsAg, the adoption of these novel therapies could potentially help them achieve functional cure in the later stages.

Furthermore, this study revealed a significant gender-based difference in HBsAg clearance, with female patients achieving higher clearance rates than males. This phenomenon has been consistently reported in multiple studies. A meta-analysis on factors influencing HBsAg seroclearance in chronic HBV patients treated with Peg-IFNα identified gender as an independent factor associated with Peg-IFN-induced HBsAg seroclearance (Jiang et al., 2024). Similarly, a real-world study investigating HBsAg clearance in HBeAg-negative CHB patients receiving Peg-IFN combination therapy also reported a gender advantage in females, with higher HBsAg seroclearance rates (Chu et al., 2022). Another large cohort study on HBeAg-negative patients treated with Peg-IFN, lamivudine, or combination therapy highlighted that baseline ALT and HBV DNA levels, patient age, female gender, and HBV genotype significantly influenced the composite response at Week 24 post-treatment (Bonino et al., 2007). However, the underlying mechanisms behind this gender difference, particularly its relationship with interferon-based therapy, remain unclear. This disparity may be attributable to certain physiological advantages of the female immune system. Studies have shown that women generally exhibit a more active immune system than men, especially in response to viral infections. Female T-cell responses are often stronger, enabling more effective viral clearance (Dunn et al., 2024). Consequently, female patients undergoing Peg-IFN therapy may benefit from more efficient immune responses, leading to better control of HBV replication and higher rates of HBsAg clearance. Additionally, sex hormones, particularly estrogen, have been demonstrated to play a critical role in modulating immune responses. Estrogen has been shown to enhance the immune functions of both B cells and T cells, which may provide the biological basis for the superior antiviral treatment outcomes observed in female patients (Hoffmann et al., 2023). While it is well-documented that women generally exhibit stronger immune responses compared to men, particularly in response to viral infections, the exact mechanisms behind this phenomenon remain unclear. We have speculated that the higher functional cure rates observed in female patients may be partly due to enhanced T-cell responses or other aspects of the female immune system. However, it is important to note that these observations are based on the existing literature and require further investigation to confirm the underlying mechanisms to better understand this potential immune advantage in women.

Previous retrospective studies have reported the occurrence of thyroid antibodies and thyroid dysfunction in 7.3% and 8.8% of patients, respectively, during Peg-IFNα therapy, with a higher prevalence in female patients (Liu et al., 2023). However, thyroid antibodies and dysfunction are not absolute contraindications for Peg-IFNα therapy, and the development of thyroid dysfunction during treatment has been linked to higher rates of HBsAg seroclearance in CHB patients (Luo et al., 2021). This phenomenon may be closely related to the immunomodulatory effects of thyroid hormones. Studies have shown that thyroid hormones, particularly FT3 and FT4, significantly influence the functions of immune cells, especially T and B cells, by promoting their proliferation and activity, thereby enhancing the immune response (Rubingh et al., 2020). This could explain the stronger ability of the immune system to clear HBV in patients with higher thyroid function, contributing to improved HBsAg clearance rates. In clinical practice, monitoring and managing thyroid function are critical for CHB patients undergoing interferon therapy. While patients with thyroid dysfunction may exhibit higher rates of HBsAg clearance, it is essential to closely monitor changes in thyroid function during treatment to avoid potential side effects or excessive immune responses. Although the exact mechanisms by which FT3 and FT4 influence HBsAg clearance remain unclear, this finding suggests that future research should further explore the potential relationship between thyroid function and the response to antiviral therapy, providing more insights for individualized treatment strategies.

The identification of baseline predictors of functional cure, such as HBsAg levels, HBeAg status, sex, and thyroid hormone levels, provides important insights for the development of personalized treatment strategies and predictive models for chronic hepatitis B (CHB). In our study, patients with HBsAg levels <100 IU/mL have a significantly higher probability of achieving functional cure within a defined treatment period, and a faster decrease in HBsAg levels was observed in patients of the functional cure group. This suggests that HBsAg quantification can therefore be used to screen patients suitable for interferon therapy and to optimize the duration and intensity of treatment (Fan et al., 2024). HBeAg-negative as well as female patients have a higher cure rate and therefore can be prioritized for treatment with interferon. In addition, in our cohort, these interferon-treated patients had thyroid function in the normal range at baseline, yet the correlation of normally high FT3 and FT4 levels with functional cure suggests a role for thyroid function in the immune response. By integrating baseline HBsAg levels and rate of decline on treatment, HBeAg status, gender, and thyroid hormone levels, clinicians can identify patients who are more likely to respond to interferon therapy and thus tailor treatment plans accordingly. Our next studies will consider integrating these baseline metrics into predictive models, which will help to develop individualized treatment regimens, improve treatment success, and reduce unnecessary treatments.

Despite the large sample size and extended follow-up period of this study, several limitations should be acknowledged. First, the inherent constraints of retrospective studies may introduce potential biases. Second, variability in patient adherence to treatment during the follow-up period might have influenced the assessment of functional cure rates. Third, while several factors have been identified as predictors of interferon response in chronic hepatitis B (CHB), such as baseline HBsAg levels and HBeAg status, HBV genotype (e.g., genotype B *vs*. C) is also a known determinant of treatment outcomes. Unfortunately, genotype data were not available for the majority of patients in this study; the lack of genotype data prevents us from assessing its potential impact on the functional cure rates in our cohort. Thus, larger-scale prospective studies are needed in the future to validate the reliability of these predictive factors and to further explore strategies for optimizing individualized treatment.

In conclusion, this study demonstrates that in chronic hepatitis B patients treated with interferon, lower baseline HBsAg levels, HBeAg negativity, female gender, and higher thyroid function are associated with an increased likelihood of HBsAg seroclearance, suggesting a better response to interferon therapy in these patients. These findings not only contribute to optimizing clinical treatment decisions but also offer new insights into achieving functional cure in chronic hepatitis B.

## Data Availability

The original contributions presented in the study are included in the article/**Supplementary Material**. Further inquiries can be directed to the corresponding author.
