# Supplementary material for: Determinants of functional cure in interferon-treated chronic hepatitis B: a retrospective cohort analysis of HBsAg dynamics and clinical predictors
Source: Front Cell Infect Microbiol. 2025 Jun 20;15:1615327. doi: 10.3389/fcimb.2025.1615327 (PMC12226575; doi:10.3389/fcimb.2025.1615327)
Supplement: Supplementary file 1 [file Table1.docx]

Supplementary table 1. Missing Data at Baseline

| Characteristics | Missing cases | Missing rates |
| --- | --- | --- |
| HBsAg (IU/mL) | 2279 cases, qualitative units (e.g., COI) accounted for 1,822 cases | 43% |
| HBV DNA (IU/mL) | 1786 cases | 33.8% |
| Imagings (Includes ultrasound, CT, MRI) | 389 cases | 7% |
| Blood biochemistry | 203 cases | 3.8% |
| Thyroid functions | 2298 cases | 43% |
| Routine blood tests | 258 cases | 4.9% |
